# Ultrashort Echo Time Double Echo Steady-State MRI for Quantitative Conductivity Mapping in the Knee: A Feasibility Study

**DOI:** 10.3390/tomography12020018

**Published:** 2026-02-02

**Authors:** Sam Sedaghat, Jin Il Park, Eddie Fu, Youngkyoo Jung, Hyungseok Jang

**Affiliations:** 1 Department of Radiology, University Hospital Heidelberg, 69120 Heidelberg, Germany; samsedaghat1@gmail.com; 2 Department of Radiology, University of California, Davis, Sacramento, CA 95817, USA; jiipark@health.ucdavis.edu (J.I.P.); eyjfu@health.ucdavis.edu (E.F.); yojung@health.ucdavis.edu (Y.J.)

**Keywords:** electric property, electric conductivity, double echo steady state, ultrashort echo time, musculoskeletal, knee, osteoarthritis, sodium, MR

## Abstract

Background/Objectives: Tissue conductivity reflects ionic composition (e.g., sodium), providing critical insights into various diseases. Ultrashort echo time quantitative conductivity mapping (UTE-QCM) offers a method to obtain this information, which is particularly effective for musculoskeletal (MSK) tissues with short T2 relaxation times. The aim of this study is to develop a UTE-QCM framework using ultrashort echo time double echo steady-state (UTE-DESS) and validate its feasibility in the knee. Methods: An ultrashort echo time double echo steady-state (UTE-DESS) sequence was used to acquire S+ and S− images and estimate the transmit radiofrequency field (B1^+^) phase at 3T. The B1^+^ phase was derived by canceling the phase evolution in the free induction decay using these images. This phase data was then processed using two widely used QCM reconstruction methods for comparison: parabolic fitting and an integral-based method. The proposed UTE-QCM framework was validated using a phantom containing three different concentrations of sodium chloride (0%, 0.5%, and 1%). Additionally, three healthy volunteers were recruited to validate UTE-QCM in knee imaging. Results: In both phantom and in vivo experiments, the integral-based QCM demonstrated improved robustness to noise compared to parabolic fitting. In the sodium phantom, the estimated conductivity showed high linearity with sodium concentrations. In the in vivo knee, the generated conductivity maps successfully visualized both long and short T2 tissues. Conclusions: We demonstrated the feasibility of UTE-QCM as a novel quantitative imaging tool targeting short T2 tissues in the MSK system. This technique may facilitate the diagnosis and prognosis of joint disorders.

## Simple Summary

Tissue conductivity reflects the amount and movement of ions, such as sodium (most abundant in the human body), which are important for tissue health. Measuring conductivity can provide valuable information about joint and musculoskeletal disorders. In this study, we developed a novel imaging method using ultrashort echo time MRI to achieve conductivity mapping in the knee. This approach successfully visualized distinct conductivity values in a phantom and healthy volunteers. The technique could help doctors better detect and monitor joint diseases, like osteoarthritis, and may guide future research into new diagnostic tools for musculoskeletal conditions.

## 1. Introduction

Electrical conductivity is a fundamental property of tissues, reflecting the density and mobility of ions transported through the material under an applied electric field. In biological tissues, this parameter is closely associated with cellular structure and ionic content. Conductivity is not a fixed value; rather, it depends on the frequency of the applied electric field [1]. At low frequencies (~100 Hz), α-dispersion dominates, where both tissue structure and composition play significant roles. In contrast, at high frequencies (~100 MHz), conductivity becomes primarily a function of tissue composition, such as ionic content in extracellular and intracellular spaces, while structural properties have minimal influence (δ-dispersion).

Recently, a novel technique called quantitative conductivity mapping (QCM) has been developed, which leverages the B1 field in magnetic resonance imaging (MRI) system [2]. This method does not require additional hardware, as it uses phase information of transmit radiofrequency field (B1^+^) encoded in MR images. At MRI frequencies (e.g., 127.7 MHz at 3T), tissues exhibit δ-dispersion, where conductivity predominantly reflects ionic composition, such as sodium (Na^+^) [3]. Because sodium plays an essential role in the human body, estimating tissue conductivity can provide critical insights into metabolic and pathological changes. QCM has been explored as a means to measure tissue conductivity, showing particular promise in the brain [4,5,6,7,8] and breast [9,10].

In the knee, sodium acts as a critical counter-ion to negatively charged glycosaminoglycans (GAGs), helping maintain the fixed charge density (FCD) and osmotic gradients essential for cartilage stiffness [11]. In osteoarthritis, early proteoglycan depletion leads to sodium loss and disruption of this electrochemical balance, suggesting that QCM could serve as a sensitive biomarker for early cartilage degeneration [12]. Specifically, the loss of negatively charged GAGs reduces the overall FCD. This decrease in FCD results in fewer available binding sites for the mobile sodium ions, causing a measurable decrease in the sodium concentration (Na^+^) within the cartilage matrix (and in other soft tissues).

To directly quantify sodium in the joint, sodium-23 (^23^Na) MRI (^23^Na-MRI) has emerged as a promising technique [13]. Since ^23^Na-MRI directly measures the density of ^23^Na spins, a drop in the signal intensity directly reflects the extent of GAG depletion, offering a non-invasive way to track disease progression. However, ^23^Na-MRI has several limitations. First, it requires a specialized radiofrequency (RF) coil for transmission and reception tuned to the ^23^Na resonance frequency at roughly 1/4 the frequency of hydrogen. Second, ^23^Na is far less abundant than ^1^H, roughly 10,000 times fewer sodium nuclei available to generate a signal compared to hydrogen protons, resulting in much lower signal levels than conventional proton MRI. Additionally, the short T2 component of sodium (intracellular sodium) is often 0.5 to 3 ms, whereas hydrogen T2 in soft tissues is typically 40 to 100 ms, producing inherently low signal-to-noise ratio (SNR). To compensate for the low SNR, extensive signal averaging is needed, leading to long acquisition times and reduced spatial resolution. These factors significantly limit the clinical utility of ^23^Na-MRI.

In contrast, QCM offers high-resolution imaging of Na^+^ and can be implemented on a clinical ^1^H MRI system without the requirement of special hardware. Current QCM utilizes conventional Cartesian imaging techniques, such as spin echo or steady-state free precession (SSFP), which cannot resolve the signal from tissues with a short T2 relaxation time in the joint, including the meniscus, ligaments, and tendons. As an alternative, in this study we explored ultrashort echo time (UTE)-based QCM (UTE-QCM) in the human knee, utilizing a UTE double echo steady-state (UTE-DESS) sequence [14,15] for the first time. The feasibility of UTE-DESS-based QCM was validated in a sodium phantom and three healthy volunteers.

## 2. Materials and Methods

### 2.1. QCM

From Maxwell’s equations, assuming that tissues are spatially homogeneous and that the gradient of the B1^+^ phase is much smaller than the change in the B1^+^ magnitude, the conductivity (σ) of the target tissue can be calculated by applying a Laplacian operator to the B1^+^ phase, $\phi^{+}$ [2]:

$$\sigma =\frac{\nabla^{2}\phi^{+}}{\omega \mu_{0}}$$

where ω represents the Larmor frequency, and $\mu_{0}$ denotes the magnetic permeability in free space. The measured B1^+^ phase is commonly preprocessed with bilateral filtering to suppress noise. However, this approach remains highly susceptible to noise because the Laplacian operator involves second derivatives. In this study, we employed the two commonly used QCM approaches: parabolic fitting and integral-based methods.

In the parabolic fitting method, $\phi^{+}$ within a kernel is fitted to a 3D second-order polynomial [9]:

$$P\left(x,y,z\right)=ax^{2}+by^{2}+cz^{2}+dxy+eyz+fxz+gx+hy+iz+j$$

The second derivative of the estimated P(x,y,z), which is 2(*a + b + c*), represents the curvature of the parabolic function and is proportional to σ. By scaling this curvature by $\omega \mu_{0}$, σ can be calculated. To satisfy the piecewise homogeneity assumption, which is a key requirement of QCM, local data within a 3D kernel are used to estimate curvature in a sliding-window manner. Additionally, tissue segmentation is often applied to enhance homogeneity within the kernel. In this study, the following processing steps were employed for parabolic fitting:

- Bilateral filtering: A bilateral filter with degree of smoothing of 3 and spatial sigma of 1 was used to reduce spatial noise in $\phi^{+}$.
- Sliding window: Data were retrieved using a sliding kernel of size 8 × 8 × 8 mm^3^.
- Outlier removal based on signal intensity: Voxels with intensity differing by more than 20% from the mean were removed.
- Parabolic fitting: Local curvature was estimated using the 3D second-order polynomial.

An alternative approach uses an integral form [16]:

$$\sigma =\frac{\int_{S}\nabla^{2}\phi^{+}dS}{V\omega \mu_{0}}$$

where S is the closed surface of a kernel with volume V. This method provides greater robustness against noise [4,17]. The processing scheme is similar to that used in parabolic fitting, employing a sliding window; however, tissue segmentation is applied in this approach to further enhance homogeneity.

The processing code was developed in MATLAB 2024b (MathWorks, Inc., Natick, MA, USA). The parabolic fitting method was implemented by our group, while the integral-based method was performed using open-source code available online (URL: https://xip.uclb.com/i/software/MRI_conductivity.html, accessed on 30 October 2024) [17].

### 2.2. UTE-QCM Using UTE-DESS

**Figure 1:**
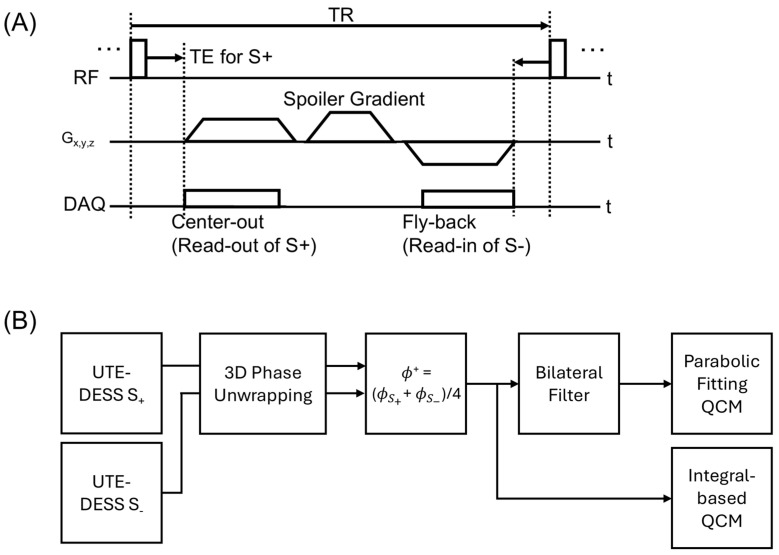
UTE-QCM. (**A**) Pulse sequence diagram of 3D radial-based UTE-DESS and (**B**) the framework of UTE-QCM. The phase maps of the S+ and S− images from UTE-DESS were first phase-unwrapped and subsequently used for calculation of the B1^+^ phase. The resulting B1^+^ phase was then processed using two QCM methods: Parabolic fitting-based QCM and integral-based QCM. For the parabolic fitting-based QCM, a bilateral filter was applied to reduce noise in the B1^+^ phase maps (RF: radiofrequency, DAQ: data acquisition, G_x,y,z_: x, y, and z gradients).

UTE-DESS is based on the steady-state of transverse magnetization similar to SSFP, but the signal is separated into S+ and S− using a spoiler gradient. In UTE-DESS, the B1^+^ phase can be estimated by averaging S− and S+ phases [14]. Figure 1A shows the pulse sequence diagram of 3D radial-based UTE-DESS, while Figure 1B illustrates the framework of the proposed UTE-QCM. To obtain the B1^+^ phase, the phase maps from S+ and S− (i.e., $\phi_{S+}$ and $\phi_{S-}$) were first unwrapped using a 3D using a region growing algorithm provided by the MEDI Toolbox (URL: https://pre.weill.cornell.edu/mri/pages/qsm.html, accessed on 1 April 2020) [18]. These phase maps were then averaged to cancel B_0_ inhomogeneity and chemical shift-induced phase, leaving only B1 phase. This is possible because of time-reversed signal formation of S−, leaving only B1 phase (i.e., combination of B1^+^ and B1^−^ phases). The B1^+^ phase was assumed to be half of the total B1 phase [19], expressed as $\phi^{+}=(\phi_{S+}+\phi_{S-})/4$. The resulting B1^+^ phase was processed using the two QCM methods described above: the parabolic fitting-based QCM and integral-based QCM.

### 2.3. Experimental Setup

The proposed UTE-QCM framework was validated using a phantom containing three different concentrations of sodium-chloride (NaCl) (0%, 0.5%, and 1% weight/volume) in distilled water, doped with 0.5 mM manganese-chloride (MnCl_2_) to shorten T2. The solutions were filled in 30 mL syringes and embedded in a large plastic container filled with 1% agarose gel.

Additionally, three healthy volunteers (37-year-old, 41-year-old, and 41-year-old males) were recruited to validate UTE-QCM in knee imaging. All subjects provided written consent under the approval of institutional review board.

Three-dimensional radial UTE-DESS MRI was conducted on a 3T clinical MRI scanner (MAGNETOM Prisma^fit^, Siemens Healthineers, Forchheim, Germany). The imaging parameters for the phantom were as follows: A receive-only 20-channel head coil, flip angle = 20°, repetition time (TR) = 5 ms, echo time (TE) of S+ = 0.08 ms, field-of-view (FOV) = 200 × 200 × 200 mm^3^, matrix size = 400 × 400 × 400, readout bandwidth (rBW) = 161 kHz, and scan time = 6 min 40 s. The imaging parameters for the human subjects were as follows: A receive-only 4-channel knee coil, FA = 20°, TR = 5 ms, TE of S+ = 0.08 ms, FOV = 200 × 200 × 192 mm^3^ (axial) or 200 × 200 × 144 mm^3^ (sagittal), matrix size = 400 × 400 × 96 (axial) or 400 × 400 × 72 (sagittal), rBW = 161 kHz, and scan time = 6 min 40 s.

## 3. Results

**Figure 2:**
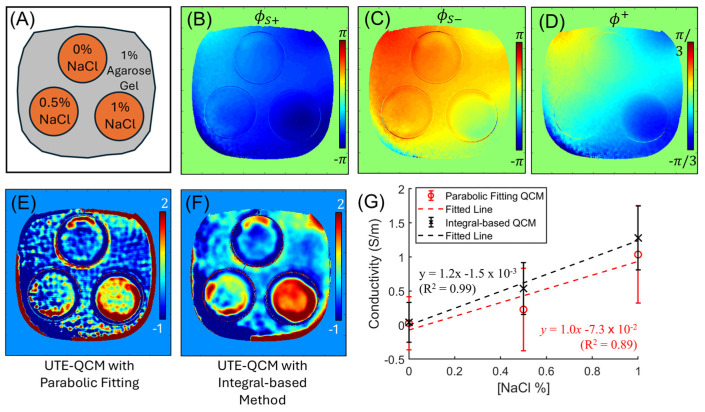
Phantom experiment. (**A**) Phantom design, (**B**) S+ phase and (**C**) S− phase maps from UTE-DESS, (**D**) the estimated B1^+^ phase, the estimated conductivity map from (**E**) parabolic fitting and (**F**) integral-based method, and (**G**) the association of mean conductivity values with the sodium concentrations (data points represent the mean conductivity values, with error bars indicating the standard error). The estimated conductivity values show high linearity to the sodium concentrations.

Figure 2 shows the estimated B_1_^+^ phase and the resulting conductivity maps using the two different QCM processing methods in the NaCl phantom. Of the two QCM approaches, the integral-based method demonstrated better noise-robustness compared to the parabolic fitting method using the second derivative of the fitted function. The estimated conductivity values in the three tubes were 0.028 ± 0.39, 0.24 ± 0.60, and 1.04 ± 0.71 S/m from the parabolic fitting-based QCM, and 0.040 ± 0.29, 0.54 ± 0.38, and 1.28 ± 0.47 S/m from the integral-based QCM, both of which showed high linearity with sodium concentrations (R^2^ = 0.89 and 0.99, respectively).

**Figure 3:**
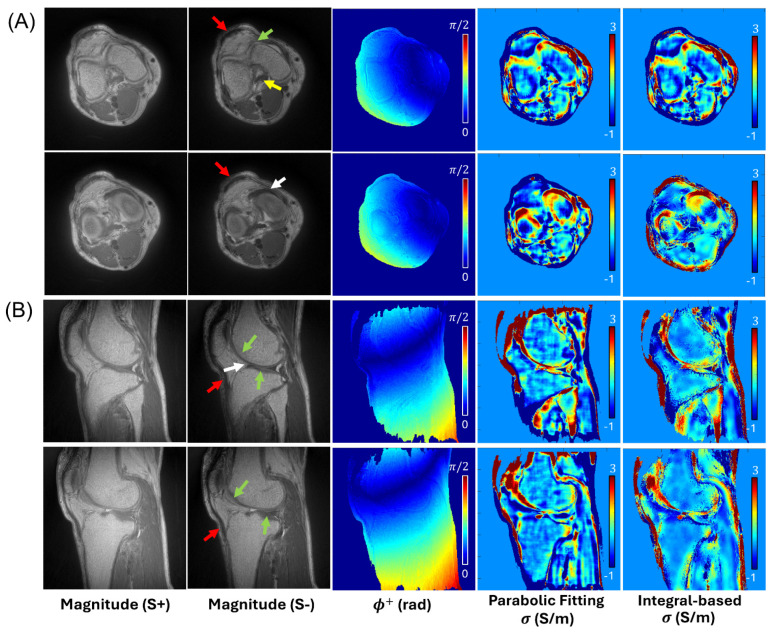
In vivo knee imaging with a healthy volunteer (42-year-old male). Two representative slices from (**A**) axial and (**B**) sagittal UTE-DESS imaging. UTE-QCM estimates conductivity in both long and short T2 tissues, including cartilage (green arrows), ligament (yellow arrow), and meniscus (white arrows), while tendon shows unreliable conductivity values (red arrows).

**Figure 4:**
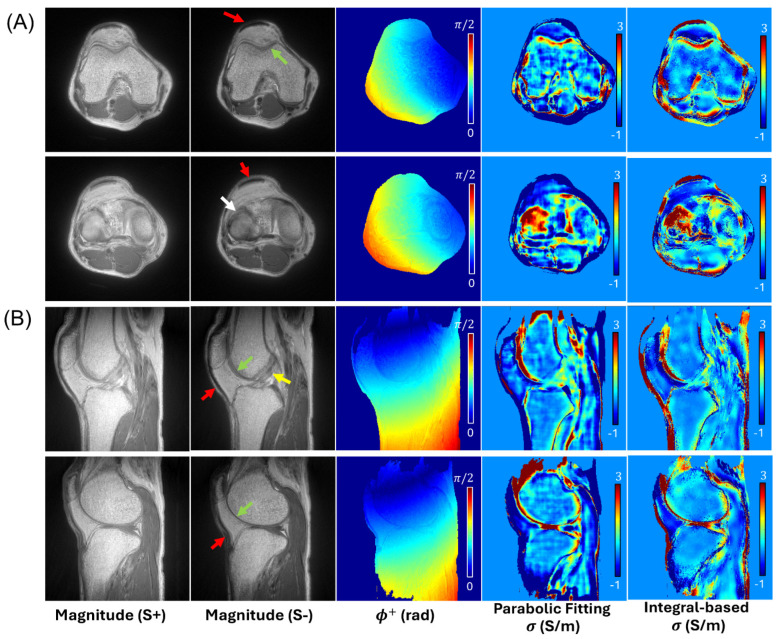
In vivo knee imaging with another healthy volunteer (42-year-old male). Two representative slices from (**A**) axial and (**B**) sagittal UTE-DESS imaging are shown. UTE-QCM produces a conductivity map with estimated values within a reasonable range (below 3 S/m), detecting both long and short T2 tissues, including cartilage (green arrows), ligament (yellow arrow), and meniscus (white arrow), but excluding tendon (red arrows).

For all subjects, UTE-DESS and the subsequent UTE-QCM yielded reasonable estimates of $\phi^{+}$ and conductivity in the knee joints. Figure 3 and Figure 4 showcase the UTE-QCM results from the two representative volunteers (two 42-year-old males), processed with the two different QCM approaches. Although both QCM methods produced conductivity estimates with visual similarity, the integral-based method demonstrated improved noise-robustness. The tissues of interest such as cartilage (green arrows), meniscus (white arrows), and ligament (yellow arrows) were detected by UTE-QCM. In tissues with extremely short T2 [20], such as the tendon, conductivity was unreliable, displaying values that were either too high or too low (red arrows). This is likely due to its anatomical location near tissue boundaries, insufficient SNR, and small size. Overall, in both QCM approaches, the estimated conductivity values in each tissue ranged ~1.5–2.5 S/m in cartilages, ~2–3 S/m in menisci, and ~0.2–0.3 S/m in ligaments.

## 4. Discussion

In this study, we demonstrated the feasibility of UTE-DESS-based QCM in the knee joint for the first time. To verify that the S+ and S− signal phases in UTE-DESS can provide B1^+^ phase information, we performed a sodium phantom experiment, which confirmed the quantitative detectability of the proposed method. In human subjects, QCM produced conductivity maps that differentiated tissue types with distinct values, demonstrating its capability for in vivo conductivity mapping. In addition, we evaluated two QCM methods and found that the integral-based approach outperformed parabolic fitting, primarily due to its enhanced noise robustness. It is well-established that standard differential approaches, such as parabolic fitting, rely on calculating the Laplacian of the phase data—a process that inherently amplifies high-frequency noise. In contrast, the integral-based formulation utilizes integration, which effectively functions as a low-pass filter, suppressing random fluctuations in the phase data. Consequently, this method maintains higher fidelity in regions with low SNR and near tissue boundaries, where differential methods are prone to generating significant edge artifacts due to signal discontinuities. Therefore, the integral-based approach is expected to provide a more stable and accurate reconstruction of conductivity maps in the musculoskeletal (MSK) system.

Conventional spin-echo-based QCM often suffers from low SNR in tissues with short T2, which is critical for musculoskeletal applications [21]. Gradient-echo imaging with variable TEs is an alternative; however, phase terms induced by chemical shift and B0 inhomogeneity complicate phase estimation at zero TE. This is particularly problematic in the MSK system, where abundant adipose and connective tissues with high magnetic susceptibility make signal fitting more complex and error-prone. Moreover, acquiring multiple TEs increases scan time and introduces potential motion artifacts. In contrast, the proposed UTE-DESS approach significantly shortens TE and requires only a single acquisition to obtain the B1^+^ phase without complex signal fitting.

One technical challenge arises at tissue boundaries. QCM assumes homogeneous tissue, an assumption often violated in real tissues, especially at boundaries. To mitigate this effect, tissue segmentation was applied. However, segmentation reduces the data available for fitting, potentially decreasing the robustness of conductivity estimation. Another challenge in UTE-DESS-based QCM is the T2-weighting effect in the S− image. Since the S− signal arises from multiple echo pathways, including spin and stimulated echoes, it is naturally more T2-weighted than S+. This can adversely affect QCM in MSK tissues with extremely short T2, due to the resulting low SNR. Furthermore, the scan time for the in vivo protocol in this study was 6 min and 40 s. This duration remains excessive for clinical imaging, which typically targets 2–3 min per sequence. To address this, more advanced view-ordering and image reconstruction techniques [22] can be applied, where a 2× acceleration can be readily achieved.

Another important consideration for MSK imaging is fat, as adipose tissue is widely present within joints and bone marrow. Because the electrical conductivity of fat is lower than that of other tissues, it may act as a confounding factor when quantifying ionic content. Building on this feasibility study, we will further explore methods for fat suppression using either a single-point Dixon approach [23] or a water-excitation method [24]. Fat–water decomposition using single-point Dixon is highly susceptible to errors such as B0 inhomogeneity and noise, which can introduce bias and corrupt the resulting data. Given that QCM is inherently prone to low SNR, additional optimization will be necessary. Water-excitation approaches have also been used for DESS imaging [25,26]. The primary drawback, however, is that RF deadtimes—both from receive-to-transmit mode switching and from transmit-to-receive switching—can be long due to the use of extended composite pulses. This limitation may be mitigated by using hard-pulse pairs, though at the expense of reduced fat-suppression performance.

It should be noted that while the integral-based QCM method employed explicit tissue segmentation, the parabolic fitting algorithm was implemented without a distinct segmentation step, adhering to the standard protocol established by Shin et al. [9]. Although this presents a procedural difference, the parabolic fitting utilizes a magnitude-based thresholding scheme that functions analogously to segmentation. By filtering data based on signal intensity, this thresholding effectively delineates the region of interest and enforces local, piecewise homogeneity similar to the masks used in the integral approach. Consequently, despite the algorithmic variance in how tissue boundaries were defined, both methods operated under comparable spatial constraints, ensuring that the observed differences in noise robustness are attributable to the reconstruction kernels rather than discrepancies in masking.

A fundamental challenge in the validation of high-frequency conductivity mapping is the absence of ground truth. Direct validation against dielectric probe measurements is often complicated by the frequency and temperature dependence of tissue properties and variability in measurement conditions [27]. Consequently, rather than relying on absolute conductivity values, this study focused on validating the method’s sensitivity to relative conductivity differences. By utilizing phantoms with systematically varied sodium concentrations, we demonstrated a strong linear relationship between the UTE-QCM derived values and the ionic concentration. This linearity confirms the internal consistency of the proposed method and its ability to accurately reflect conductivity changes, which is a critical step in verifying the technique’s utility for clinical applications where in vivo ground truth is unavailable.

The clinical relevance of UTE-QCM lies in its ability to detect ionic alterations in tissues that are central to joint disease. Cartilage degeneration in osteoarthritis begins with early proteoglycan depletion, which reduces sodium content and thereby lowers conductivity [13,28,29]. The ability of UTE-QCM to generate conductivity maps of cartilage, meniscus, and ligaments using standard hardware suggests that this technique could serve as an early biomarker of biochemical tissue degradation, preceding morphologic changes visible on conventional MRI. Furthermore, integrating conductivity information with other quantitative UTE MRI techniques [30] may enhance diagnostic confidence and enable more personalized monitoring of disease progression or therapeutic response.

This feasibility study has several limitations. While this study successfully demonstrates the initial feasibility and potential of using UTE-DESS for QCM in the knee, we acknowledge certain limitations inherent to a proof-of-concept investigation. Our primary objective was to establish that the UTE-DESS sequence could generate physiologically consistent conductivity maps in complex joint environments, a capability that has not been previously reported. Consequently, comprehensive evaluations, such as extensive scan-rescan repeatability testing, inter-scanner comparisons, and a rigorous analysis of signal dependencies on B1^+^ inhomogeneity and T2 relaxation, were beyond the scope of this preliminary work. Additionally, the validity of conductivity assumptions in thin knee joint tissue structures and potential boundary effects require further characterization. Furthermore, we did not compare UTE-DESS-based QCM with other MR sequences, such as SSFP or spin-echo, which are expected to yield lower SNR and more degraded QCM estimates. We anticipate that those conventional sequences will be less sensitive to short T2 tissues due to the long TE. Lastly, we did not validate our results against ^23^Na-MRI, which could provide ground-truth imaging information to assess the quantitative accuracy of UTE-QCM. Future studies will recruit cohorts of patients with degenerative joint disorders, such as osteoarthritis, as well as healthy controls, to investigate the diagnostic potential of this approach. These follow-up investigations will focus on establishing the quantitative accuracy, precision, repeatability, and reproducibility necessary to translate this technique into routine clinical practice.

## 5. Conclusions

In this study, we demonstrated for the first time the feasibility of QCM using UTE-DESS. Further optimization and validation of this technique in clinical settings may confirm its diagnostic potential as a sensitive, quantitative imaging tool for assessing ionic changes in joint tissues in degenerative disorders and other musculoskeletal conditions.

## Data Availability

The original contributions presented in this study are included in the article. Further inquiries can be directed to the corresponding author.

## Abbreviations

The following abbreviations are used in this manuscript:

UTE-QCM	Ultrashort Echo Time Quantitative Conductivity Mapping
MSK	Musculoskeletal
UTE-DESS	Ultrashort Echo Time Double Echo Steady-State
B1^+^	Transmit Radiofrequency Field
QCM	Quantitative Conductivity Mapping
MRI	Magnetic Resonance Imaging
GAG	Glycosaminoglycan
FCD	Fixed Charge Density
^23^Na	Sodium-23
RF	Radiofrequency
^1^H	Proton
SNR	Signal-To-Noise Ratio
SSFP	Steady-State Free Precession
UTE	Ultrashort Echo Time
S+/S−	Positive and Negative DESS Echoes
NaCl	Sodium Chloride
TE	Echo Time
TR	Repetition Time
FOV	Field of View
rBW	Readout Bandwidth
